# SARS-CoV-2-Specific Adaptive Immunity in COVID-19 Survivors With Asthma

**DOI:** 10.3389/fimmu.2022.947724

**Published:** 2022-07-18

**Authors:** Li Chen, Junqing Yue, Shengding Zhang, Wenxue Bai, Lu Qin, Cong Zhang, Bihao Wu, Moxuan Li, Shuyun Xu, Qing Jiang, Lin Yang, Qingxiu Xu, Rongfei Zhu, Min Xie, Rui Gong

**Affiliations:** ^1^ CAS Key Laboratory of Special Pathogens and Biosafety, Wuhan Institute of Virology, Center for Biosafety Mega-Science, Chinese Academy of Sciences, Wuhan, China; ^2^ University of Chinese Academy of Sciences, Beijing, China; ^3^ Department of Respiratory and Critical Care Medicine, Tongji Hospital, Tongji Medical College, Huazhong University of Science and Technology, Wuhan, China; ^4^ Key Laboratory of Respiratory Diseases, National Ministry of Health of the People’s Republic of China and National Clinical Research Center for Respiratory Disease, Wuhan, China; ^5^ Department of Allergy, Tongji Hospital, Tongji Medical College, Huazhong University of Science and Technology, Wuhan, China

**Keywords:** asthma, COVID-19, immune memory, neutralizing antibodies, T cell responses

## Abstract

**Background:**

Asthma patients potentially have impaired adaptive immunity to virus infection. The levels of SARS-CoV-2-specific adaptive immunity between COVID-19 survivors with and without asthma are presently unclear.

**Methods:**

COVID-19 survivors (patients with asthma n=11, with allergies n=8, and COVID-19 only n=17) and non-COVID-19 individuals (asthmatic patients n=10 and healthy controls n=9) were included. The COVID-19 patients were followed up at about 8 months and 16 months after discharge. The clinical characteristics, lymphocyte subsets, memory T cells, and humoral immunity including SARS-CoV-2 specific antibodies, SARS-CoV-2 pseudotyped virus neutralization assay, and memory B cells were analyzed in these subjects.

**Results:**

The strength of virus-specific T cell response in COVID-19 survivors was positively correlated with the percentage of blood eosinophils and Treg cells (r=0.4007, p=0.0188; and r=0.4435, p=0.0086 respectively) at 8-month follow-up. There were no statistical differences in the levels of SARS-CoV-2-specific T cell response between the COVID-19 survivors with, and without, asthma. Compared to those without asthma, the COVID-19 with asthma survivors had higher levels of SARS-CoV-2-specific neutralizing antibodies (NAbs) at the 8-month follow-up (p<0.05). Moreover, the level of NAbs in COVID-19 survivors was positively correlated with the percentage of Treg and cTfh2 cells (r=0.5037, p=0.002; and r=0.4846, p=0.0141), and negatively correlated with the percentage of Th1 and Th17 cells (r=-0.5701, p=0.0003; and r=-0.3656, p=0.0308), the ratio of Th1/Th2, Th17/Treg, and cTfh1/cTfh2 cell (r=-0.5356, r=-0.5947, r=-0.4485; all p<0.05). The decay rate of NAbs in the COVID-19 survivors with asthma was not significantly different from that of those without asthma at 16-month follow-up.

**Conclusion:**

The level of SARS-CoV-2-specific NAbs in COVID-19 survivors with asthma was higher than that of those without asthma at 8-month follow-up. The SARS-CoV-2-specific T cell immunity was associated with blood eosinophils and Treg percentages. The SARS-CoV-2-specific humoral immunity was closely associated with cTfh2/cTfh1 imbalance and Treg/Th17 ratio. According to the findings, asthmatic patients in COVID-19 convalescent period may benefit from an enhanced specific humoral immunity, which associates with skewed Th2/Th1 and Treg/Th17 immune.

## Introduction

As of October 2021, the Coronavirus disease 2019 (COVID-19) pandemic has been responsible for more than 4.6 million deaths worldwide (1). Disease severity ranges from asymptomatic through to fatal, and data from several studies has suggested that older age and comorbidities, including hypertension, diabetes, and cardiovascular disease, are major risk factors for COVID-19 mortality (2–4).

Asthma is one of the most prevalent chronic airway inflammatory diseases worldwide and is closely related to type 2 immune responses (5). A variety of respiratory viruses, including rhinoviruses, the influenza virus, and coronaviruses, can affect the upper and lower airways and induce asthma attacks (6). Interestingly, existing studies have not indicated that COVID-19 exacerbates asthma or that there has been a high prevalence of asthma among COVID-19 patients (7). It has previously been observed that in most countries, asthmatic patients have usually had similar or lower rates of COVID-19 infection, rather than higher rates, when compared to general populations within the corresponding areas (4, 8–11). However, there is no clear evidence that the severity and mortality rates among COVID-19 patients with asthma are higher than for patients without asthma (12, 13).

Previously, some studies indicated that asthmatic patients may experience an exacerbated inflammatory response and may be at a higher risk of mortality after COVID-19 infection (14–16). While, in another study including COVID-19 patients from the United States, South Korea, and Europe, it was discovered that mortality in COVID-19 patients with and without asthma was similar (17). Moreover, among hospitalized patients 65 years or younger with severe COVID-19, asthma diagnosis was not associated with worse outcomes, regardless of age, obesity, or other high-risk comorbidities (18). Our previous studies showed that the prevalence of asthma in patients with COVID-19 was markedly lower than that reported in the adult population of Wuhan (0.9% vs 6.4%) (4). Overall, we speculate that asthma may be a protective factor for COVID-19 (19–21).

As far as now, there are no related report reveal whether asthma patients experience altered specific immunity against acute respiratory syndrome coronavirus 2 (SARS-CoV-2). While, Jing Li et al. identified elevated levels of KIR^+^CD8^+^ T cells, but not CD4^+^ regulatory T cells, in COVID-19 patients, which were associated with disease severity and vasculitis (22). Another previous literature showed that the stimulation of immune cells with live SARS-CoV-2 induced a rapid decline in the pool of effector memory CD8^+^, but not CD4^+^, T cells after recovery from COVID-19 (23). Moreover, Gong et al. demonstrated a close connection between CD4^+^T cells and antibody production in COVID-19 convalescent patients (24). Cellular and humoral immunity plays an important role in SARS-CoV-2 infection and clinical recovery. Moreover, SARS-CoV-2-specific T cells, B cells, and antibodies may persist more than one year after patients have recovered from SARS-CoV-2 infection and may also predict their re-infection risk (25, 26). Specific immunity in asthmatic patients after recovery from COVID-19 has rarely been reported.

Given the high incidence of both COVID-19 and asthma, and the lack of clinical studies to date, the present study aimed to investigate the levels of SARS-CoV-2-specific humoral and cellular immunity in patients with and without asthma. In addition, this research explored the relationships between these SARS-CoV-2 specific immunity levels and the baseline T lymphocyte subsets and immune polarization of these patients, as well as their clinical features and outcomes from laboratory tests.

## Methods

### Study Subjects

In total, 36 convalescing COVID-19 individuals (who also had asthma n=11, allergies n=8, or COVID-19 only n=17) and age- and sex-matched asthmatic patients (n=10) and healthy donors (n=9) who were not vaccinated with COVID-19 vaccine were recruited. The COVID-19 patients had received positive laboratory test results using the SARS-CoV-2 nucleic acid test between January 2020 to March 2020, in Wuhan, China. Subject to the word limit of the article, other materials and method content are included in the **Supplementary Material**.

## Results

### Demographic and Baseline Clinical Characteristics

Full demographic and baseline clinical characteristics of participants are detailed in **Table 1**. There were no significant differences in age, sex, or BMI among the five groups, which was expected as they had been matched for these characteristics. Th2-high asthma was found in 9 of 11 COVID-19 with asthmatic patients and 9 of 10 asthmatic patients not affected by COVID-19. The rate of severe COVID-19 was slightly higher in patients within the COVID-19 only group than patients in the COVID-19 with asthma or allergy groups (47.1% vs 9.1% vs 37.5%, p=0.114). There were no statistical differences in the underlying comorbidities between all participants, besides hypertension, which is higher in COVID-19 with asthma group (p=0.001, **Table 1**).

**Table 1:** Demographic and baseline clinical characteristics of subjects.

	COVID-19 with Asthma	COVID-19 with Allergy	COVID-19	Asthma	Healthy control	*p* value
**Number**	11	8	17	10	9	–
Age (years)	50 (36–71)	57 (34.75-70.25)	49 (38-68)	54.5 (46.75-58.75)	56 (38.5-63.5)	0.997
Gender (Female/Male)	7/4	5/3	8/9	6/4	6/3	0.874
BMI (kg/m^2^)	26.21 ± 3.75	22.77 ± 3.46	24.45 ± 2.95	25.38 ± 3.56	24.21 ± 3.93	0.282
Smoking history, n (%)	2 (18.2%)	2 (25%)	2 (11.8%)	1 (10%)	0 (0%)	0.638
Severe COVID-19, n (%)	1 (9.1%)	3 (37.5%)	8 (47.1%)	–	–	0.114
Severe asthma, n (%)	3 (27.3%)	–	–	2 (20%)	–	>0.999
Th2-high asthma, n (%)	9 (81.8%)	–	–	9 (90%)	–	>0.999
Atopy, n (%)	5 (45.5%)	8 (100%)	0 (0%)	8 (80%)	0 (0%)	**<0.001** ^*#¶§^
**Underlying comorbidity, n (%)**						
COPD	0 (0%)	0 (0%)	0 (0%)	0 (0%)	0 (0%)	–
Interstitial lung Disease	0 (0%)	0 (0%)	0 (0%)	0 (0%)	0 (0%)	–
Tuberculosis	0 (0%)	0 (0%)	0 (0%)	0 (0%)	0 (0%)	–
Bronchiectasis	0 (0%)	0 (0%)	0 (0%)	0 (0%)	0 (0%)	–
Diabetes	1 (9.1%)	0 (0%)	1 (5.9%)	0 (0%)	0 (0%)	>0.999
Hypertension	8 (72.7%)	2 (25%)	5 (29.4%)	1 (10%)	0 (0%)	**0.003** ^*¶§^
CHD	2 (18.2%)	0 (0%)	1 (5.9%)	0 (0%)	0 (0%)	0.517
Hepatitis B	0 (0%)	0 (0%)	1 (5.9%)	1 (10%)	0 (0%)	0.874
CKD	0 (0%)	0 (0%)	0 (0%)	0 (0%)	0 (0%)	–
Tumor	0 (0%)	0 (0%)	0 (0%)	0 (0%)	0 (0%)	–

Data were expressed as mean ± SD, median (interquartile range), and No. (%). Multiple groups were compared using one-way analysis of variance (ANOVA) test with Tukey intergroup comparison (normal data) or a Kruskal-Wallis test with a Dunn intergroup comparison (non-normal data). The Fisher exact tests were used to compare ratios. Bold values indicate significant differences (p<0.05).

*: COVID-19 with Asthma vs COVID-19, p < 0.05.

#: COVID-19 with Allergy vs COVID-19, p < 0.05.

¶: COVID-19 with Asthma vs COVID-19 with Allergy, p < 0.05.

§: COVID-19 with Asthma vs Asthma, p < 0.05.

BMI, body mass index; COPD, chronic obstructive pulmonary disease; CHD, coronary heart disease; CKD, chronic kidney disease.

### Clinical Characteristics, Laboratory Findings, Pulmonary Function, and Radiographic Findings at 8-Month Follow Up

Comparisons in COVID-19 patients 8 months after discharge were conducted. As shown in **Table 2**, some COVID-19 survivors still had persistent physical and (or) psychological symptoms, but there were no obvious differences between COVID-19 patients with or without asthma. The result of the 6MWD test in COVID-19 patients with asthma were slightly lower than that in the other two groups (p=0.292). Furthermore, there were no significant differences in the laboratory findings between the COVID-19 patients with either asthma, allergies, or COVID-19 only at the 8-month follow-up period.

**Table 2:** Characteristics of COVID-19 survivor with/without asthma or allergy at 8-month follow-up.

Eight months after discharge	COVID-19 with Asthma	COVID-19 with Allergy	COVID-19	*p* value
**Number**	11	8	17	*-*
**Clinical symptoms**				
PCFS scale grade≥1, n (%)	4 (36.4%)	4 (50%)	9 (52.9%)	0.689
PCFS scale grade≥2, n (%)	1 (9.1%)	2 (25%)	4 (23.5%)	0.642
**6MWD, m**	502 ± 95	549.6 ± 88.6	554.8 ± 83.7	0.292
**Laboratory data**				
WBC (× 10^9/L)	5.46 (5-6.11)	5.35 (4.61-5.86)	6.2 (4.92-7.31)	0.212
Lymphocyte count (× 10^9/L)	1.77 (1.56-2.00)	1.985 (1.53-2.38)	2.21 (1.77-2.78)	0.421
Eosinophil count (× 10^9/L)	0.2 (0.07-0.33)	0.18 (0.06-0.28)	0.13 (0.06-0.21)	0.796
T-IgE (KU/I)	134 (71.2-288)	85.4 (17.78-100)	100 (43.45-100)	0.146
**SARS-CoV-2-specific antibodies**				
IgG (S/CO)	7.82 (5.05-10.14)	6.55 (3.56-8.07)	6.31 (2.97-8.56)	0.300
IgM (S/CO)	0.2 (0.08-0.54)	0.165 (0.11-0.27)	0.23 (0.065-0.7)	0.812
**Pulmonary function**				
FEV1% predicted	91.0 ± 25.1	106.2 ± 14	104.8 ± 14.6	0.116
FVC% predicted	105.8 ± 22.9	115.1 ± 16.6	113.4 ± 17.4	0.491
FEV1/FVC, %	71.2 ± 12.0	76.8 ± 77.2	76.3 ± 6.9	0.291
MEF50% predicted	55.5 ± 27.3	84.7 ± 17.86	74.8 ± 18.5	**0.015** ^¶^
MEF25% predicted	43.2 (16.2-48.2)	57.1 (39.5-80.7)	48.1 (35.5-59.7)	0.218
MMEF75/25% predicted	49.5 ± 25.1	68.2 ± 28.5	65.5 ± 18.6	0.142
MVV% predicted	91.7 ± 16.1	111.5 ± 14.4	115.6 ± 15.6	**0.0012** ^*¶^
TLC% predicted	96.4 ± 11.5	101.6 ± 9.2	98.1 ± 6.8	0.463
DLCO% predicted	85.8 ± 13.2	83.7 ± 11.0	89.4 ± 14.9	0.586
DLCO/VA% predicted	92.0 ± 11.8	85.3 ± 14.8	93.7 ± 14.0	0.361
**Chest HRCT**				
CT score	2 (1-3)	0.5 (0-1.75)	1 (0-3.5)	0.445
Abnormal HRCT (CT score≥5), n (%)	2 (18.2%)	1 (12.5%)	3 (17.6%)	>0.999
GGO, n (%)	5 (45.5%)	3 (37.5%)	6 (35.3%)	0.905
Irregular lines, n (%)	9 (81.8%)	2 (25%)	7 (41.2%)	**0.031** ^*#¶^
Consolidation, n (%)	0 (0%)	0 (0%)	0 (0%)	–
Interlobular septal thickening, n (%)	0 (0%)	0 (0%)	0 (0%)	–
Subpleural line, n (%)	0 (0%)	0 (0%)	0 (0%)	–
Reticular pattern, n (%)	0 (0%)	0 (0%)	1 (5.9%)	>0.999
**Treatment post-discharge**				
ICS/LABA, n (%)	6 (55%)	0 (0%)	0 (0%)	<0.001^*¶^
ICS dose (BDP equivalent, ug/d)	200 (0-400)	–	–	–
Systemic glucocorticoid, n (%)	0 (0%)	0 (0%)	0 (0%)	–
LTRA, n (%)	1 (9%)	1 (12.5%)	1 (5.9%)	>0.999
ACEI/ARB, n (%)	4 (36.4%)	0 (0%)	3 (17.6%)	0.182
Antibiotics, n (%)	0 (0%)	0 (0%)	0 (0%)	–
Anticoagulants, n (%)	1 (9.1%)	0 (0%)	1 (5.9%)	>0.999
Immunosuppressive drug, n (%)	0 (0%)	0 (0%)	1 (5.9%)	>0.999
Antiviral drugs, n (%)	0 (0%)	0 (0%)	0 (0%)	–
**Time from discharge to visit 1, d**	239 ± 12	246 ± 15	245 ± 13	0.491

Data were expressed as mean ± SD, median (interquartile range), or No. (%). Multiple groups were compared using one-way analysis of variance (ANOVA) test with Tukey intergroup comparison (normal data) or a Kruskal-Wallis test with a Dunn intergroup comparison (non-normal data). Bold values indicate significant differences (p<0.05).

*: COVID-19 with Asthma vs COVID-19, p < 0.05.

#: COVID-19 with Allergy vs COVID-19, p < 0.05.

¶: COVID-19 with Asthma vs COVID-19 with Allergy, p < 0.05.

PCFS scale, post-COVID-19 functional status scale; 6MWD, six-minute walk distance; FEV1, forced expiratory volume in one second; FVC, forced vital capacity; MEF50, maximal expiratory flow at 50% of FVC; MEF25, maximal expiratory flow at 25% of FVC; MMEF75/25, maximal mid-expiratory flow between 75% and 25% of FVC; MVV, maximum voluntary ventilation; TLC, total lung capacity; DLCO, diffusion capacity of the lung for carbon monoxide; DLCO/VA, ratio of carbon monoxide diffusion capacity to alveolar ventilation; HRCT, high-resolution computed tomography; GGO, ground glass opacity; ICS/LABA, combination inhaled corticosteroids plus long-acting β-agonists; BDP, beclomethasone dipropionate; LTRA, leukotriene receptor-antagonist; ACEI/ARB, angiotensin converting enzyme inhibition/angiotensin receptor blocker.

Pulmonary function tests were completed in all of the COVID-19 patients 8 months following discharge. Compared with the other two COVID-19 groups, patients with asthma plus COVID-19 had a lower MVV% predicted (p=0.0012) and MEF50% predicted (p=0.015), which represented a reduced ventilation reserve capacity and limited small airways functions. It was found that there were no statistical differences in the CT scores between the three COVID-19 groups, however, the proportion of irregular lines on the chest CT imaging 8 months after discharge was higher in COVID-19 with asthma group than in the other two COVID-19 groups (45.5% vs 37.5% vs 35.3%, p=0.031). More detailed information is summarized in **Table 2**.

### Peripheral Blood Lymphocyte Subsets in Subjects

**Figure 1:**
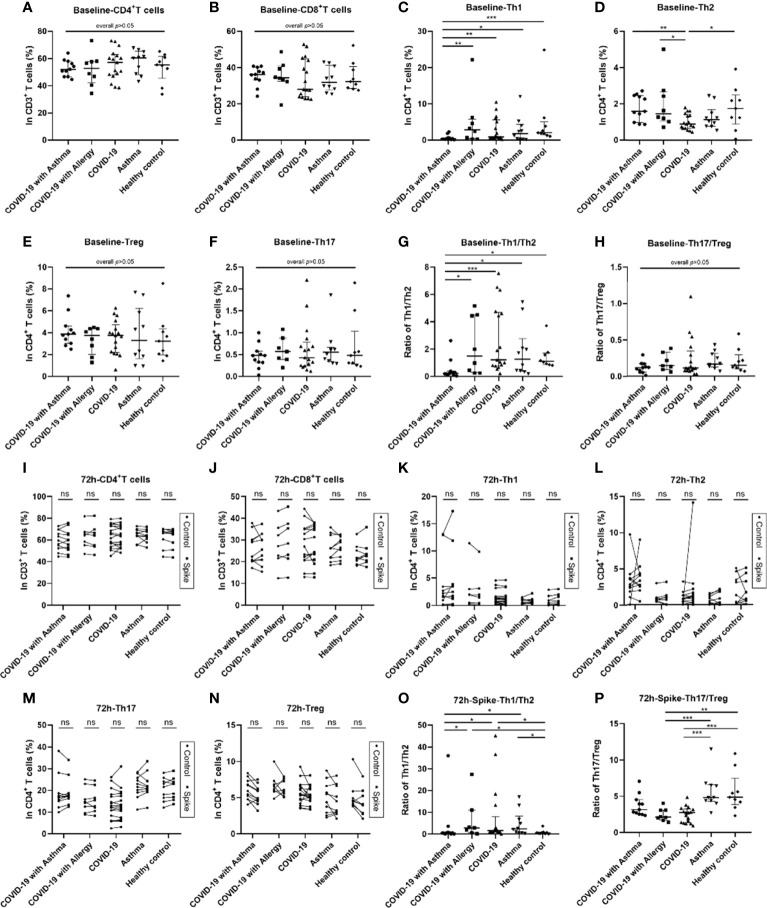
Blood lymphocyte subsets at baseline and 72 hours after SARS-CoV-2 Spike peptide pools stimulation among COVID-19 groups with/without asthma or allergy. Analysis of blood lymphocyte subsets in COVID-19 with asthma group (n = 11), COVID-19 with allergy group (n = 8), COVID-19 group (n = 17), asthma group (n = 10) and healthy control group (n = 9). Lymphocyte subsets were detected by flow cytometry for CD4^+^T cells, CD8^+^T cells, Th1 cells, Th2 cells, Th17 cells, and Treg cells at the baseline **(A–H)** and 72 hours after stimulation by SARS-CoV-2 Spike peptide pools or PBS control **(I–P)**. The percentages of and the ratios of CD4^+^/CD8^+^, Th1/Th2, and Th17/Treg in the five groups were analyzed. Data were tested using the Kruskal-Wallis test and Dunn’s test. Control and spike peptide pools stimulation groups were compared using Wilcoxon Matched-Pairs signed rank test. ^*^ *p* < 0.05, ^**^ *p* < 0.01, ^***^ *p* < 0.001, ns, not significant.

Flow cytometry was used to detect the changes in peripheral blood T lymphocyte subsets in the five groups of participants. The results are presented in **Figure 1** and **Figure E2** and the gating strategy of flow cytometry is shown in **Figure E1**. Within CD3^+^CD4^+^T lymphocytes, CD183^+^T cells, CD294^+^T cells, CD196^+^T cells, and CD25^high^CD127^low^ T cells were defined as Th1, Th2, Th17, and Treg cells, respectively.

These lymphocyte subsets were detected by flow cytometry at the baseline, 24 hours, and 72 hours after PBS control and SARS-CoV-2 Spike peptide pools (S) stimulation. The T helper cell compartment, median of baseline Th1 cells percentage, and Th1/Th2 ratio decreased significantly in COVID-19 patients with asthma, they were lower than observed in the COVID-19 only, asthma without COVID-19 and healthy control groups (all p<0.05, **Figures 1C, G**). The frequency of baseline Th2 cells in COVID-19 only group was lower than that in COVID-19 with asthma group (p<0.05, **Figure 1D**). While no significant differences were observed in the percentages of baseline CD4^+^T cells, CD8^+^T cells, Th17 cells, Treg cells, CD4^+^/CD8^+^ T cell ratio, and Th17/Treg cell ratio among the five groups (**Figures 1A, B, E, F, H**). At 24-hour, the difference in T lymphocyte subsets in the SARS-CoV-2 S stimulated group and PBS control group were similar to the baseline results (**Figures E2A, B**). Moreover, no significant differences in the proportions of CD4^+^T, CD8^+^T, Th1, Th2, Th17, or Treg cells were found in the 72 hours S stimulated group compared with the PBS control group (**Figures 1I–N**). The ratios of Th1/Th2 cells in the COVID-19 with asthma group was still lower (p=0.015) at 72 hours after S stimulation (**Figure 1O**). At the same time, the experimental observation found that the Th17/Treg ratio in the COVID-19 group was lower than that observed in the asthma group (non-COVID-19) and healthy control group after 72 hours of S stimulation (**Figure 1P**).

In addition, the proliferation of T lymphocyte subsets within the five groups were detected using CFSE assays at 72 hours after S stimulation or a PBS control. There was no obvious proliferation of T lymphocyte subsets in the peripheral blood of all subjects (**Figure E3**).

### SARS-CoV-2 Specific Memory T Cell Responses Among COVID-19 Survivors and Controls

Two methods were used to detect SARS-CoV-2 specific memory T cell responses. Intracellular cytokine (ICS) analysis was utilized to assess SARS-CoV-2-specific CD4^+^ and CD8^+^ T cells in the peripheral blood from all participants. Interferon (IFN)-γ ELISA analysis was used to determine the magnitude of the global SARS-CoV-2-specific memory T cell response.

**Figure 2:**
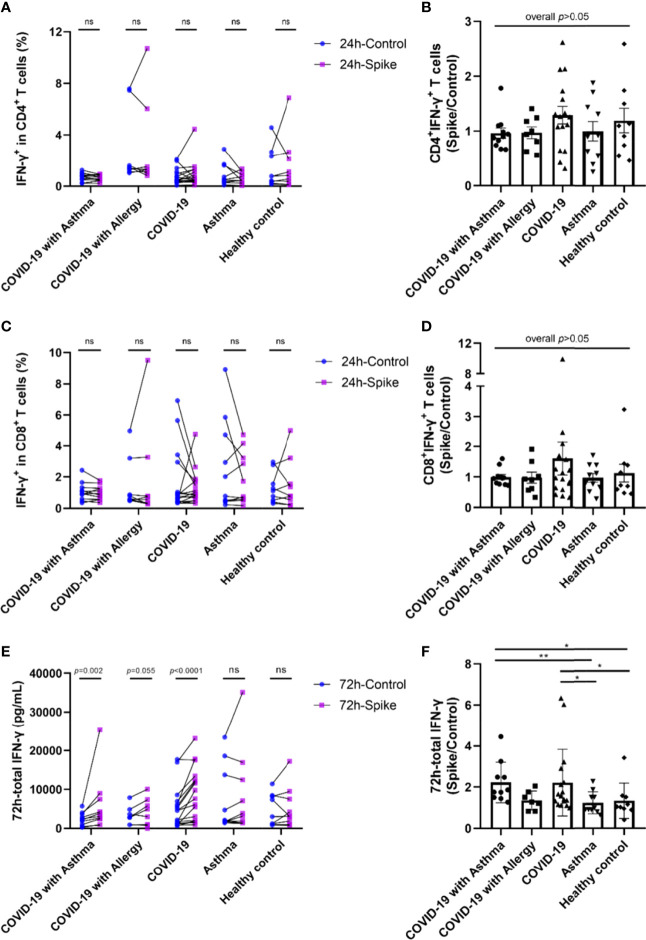
SARS-CoV-2-specific memory T cell responses among COVID-19 survivors and controls. PBMCs were stimulated with SARS-CoV-2 Spike peptide pools or PBS control. **(A, C)** IFN-γ positive ratio of CD4^+^T cells and CD8^+^T cells against spike peptide pools or PBS control were detected by intracellular cytokine staining (ICS) and flow cytometry method. **(E)** IFN-γ in the supernatant of total T cells culture measured by ELISA. **(B, D, F)** The ratio of specific T cell response by spike peptide pools stimulation compared with PBS control. Each point on the graph represents a single donor. Control and spike peptide pools stimulation groups were compared using Wilcoxon matched-pairs signed rank test. The significance between each group was determined using the Kruskal-Wallis test and Dunn’s multiple comparison test. ICS, intracellular cytokine staining. ^*^ *p* < 0.05, ^**^ *p* < 0.01, ^***^ *p* < 0.001, ns, not significant.

Although most COVID-19 survivors have specific T cell responses to SARS-CoV-2, the results from the virus-specific spike/control ratio of CD4^+^T cells and CD8^+^T cells in the five groups were not statistically significantly different (**Figures 2A–D**). However, when the 17 COVID-19 survivors were divided into 9 non-severe patients and 8 severe patients, the median ratios of spike/control IFN-γ percentages of CD4^+^ and CD8^+^ T cell in the severe COVID-19 group were higher than those in the non-severe group (**Figures E4A, B**).

IFN-γ levels were increased in the total T cell culture supernatants from COVID-19 survivors 72h after S stimulation compared to the PBS control (**Figure 2E**). Among the 11 patients in the COVID-19 with asthma group, one patient’s IFN-γ level exceeded the detected value range, and the remaining 10 patients all showed increased IFN-γ levels (p<0.01). In the COVID-19 with allergy group, 1 in 8 patients had an IFN-γ beyond the detection range, and 5 of the remaining 7 patients had IFN-γ values which increased in the spike stimulation group when compared with the PBS control group (p=0.055). IFN-γ levels also increased in 16 of the 17 patients in the COVID-19 only spike-stimulated group compared with the PBS control group (p<0.0001). There were no significant differences in IFN-γ levels between the PBS control and S stimulation in asthma (non-COVID-19) and healthy control group. No significant differences were found between the median of spike/control ratio of IFN-γ in the COVID-19 with asthma group and the COVID-19 only group (**Figure 2E**). In addition, the intracellular TNF-α, IL-4, and IL-10 were detected by flow cytometry. The percentage of TNF-α, IL-4, and IL-10 positive CD4^+^ and CD8^+^T lymphocytes showed little difference between the control group and the spike stimulation group, with no regular trend (all p<0.05, data not shown). Overall, these results indicated no significant differences in the strength of the SARS-CoV-2-specific T cell responses between patients with and without asthma at 8-month follow-up after recovery from COVID-19 (**Figure 2F**).

### SARS-CoV-2-Specific Humoral Response and Memory B Cells Among COVID-19 Survivors and Controls

**Figure 3:**
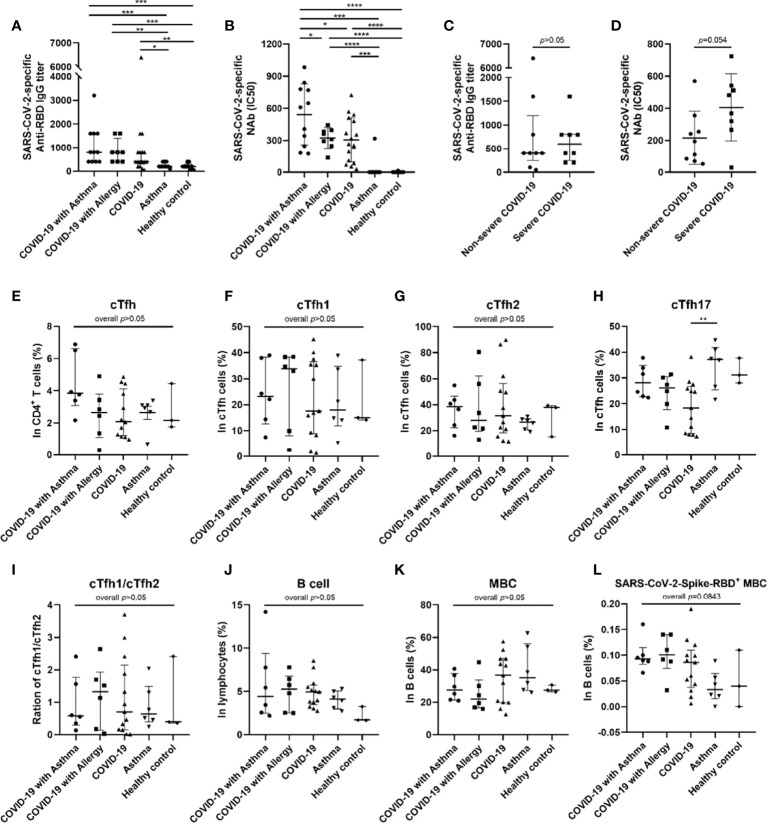
SARS-CoV-2-specific antibodies and frequency of cTfh and memory B cells in peripheral blood of subjects. Anti-SARS-CoV-2 RBD antibody titer was compared among five groups **(A)** (COVID-19 with asthma n = 11, COVID-19 with allergy n = 8, COVID-19 n = 17, asthma n = 10, healthy control n = 9). The half-maximal inhibitory concentration (IC50) of SARS-CoV-2-specific neutralizing antibodies (NAbs) was compared among five groups **(B)**. The median of anti-RBD IgG titer **(C)** and mean of IC50 **(D)** of patients recovering from severe COVID-19 (n = 8) were higher than that of patients with non-severe COVID-19 (n = 9). The frequency of cTfh cells **(E)** and B cells **(J)** were detected and compared in COVID-19 with asthma group (n = 6), COVID-19 with allergy group (n = 6), COVID-19 group (n = 13), asthma group (n = 6), and healthy control group (n = 3). **(F–I)** Frequency of cTfh1, cTfh2, and cTfh17 cells in cTfh cells was compared among different groups. **(K, L)** The percentages of memory B cells and SARS-CoV-2 RBD^+^ memory B cells were compared among the five groups. Statistical analysis was performed using Kruskal–Wallis test or Welch ANOVA tests using Graphpad Prism software. For statistical comparisons between the severe and non-severe groups, unpaired t-tests were performed. ^*^ *p* < 0.05, ^**^ *p* < 0.01, ^***^ *p* < 0.001, ^****^ *p* < 0.0001.

At the 8-month follow-up, it was found that all of the COVID-19 patients remained positive for anti-RBD IgG and NAbs. However, there was no statistically significant difference in anti-RBD IgG titers between COVID-19 groups with and without asthma (**Figure 3A**). More notably, the half maximal inhibitory concentration (IC50) value of the COVID-19 with asthma group was higher than that of the COVID-19 group without asthma (p<0.05, **Figure 3B**), which indicated that the patients in COVID-19 with asthma group had a stronger SARS-CoV-2 pseudovirus neutralization capacity than the COVID-19 without asthma group. In addition, the mean value of IC50 in severe COVID-19 only survivors was higher than that in non-severe patients (p=0.054, **Figure 3D**). There was no significant difference in anti-RBD IgG titers between the severe and non-severe COVID-19 groups (**Figure 3C**). Subsequently, the correlation of SARS-CoV-2-NAbs with total SARS-CoV-2-specific IgG and IgM were evaluated and a positive correlation was found (p<0.0001 and p<0.05, respectively; **Figures E5I, J**). It is noteworthy that there was no significant difference in the shedding time of SARS-CoV-2 between COVID-19 with and without asthma groups (p>0.05, **Figure E6A**), and there was no correlation between SARS-CoV-2-specific antibodies at the 8-month follow-up and that of during hospitalization (**Figures E6C, D**).

**Figure 4:**
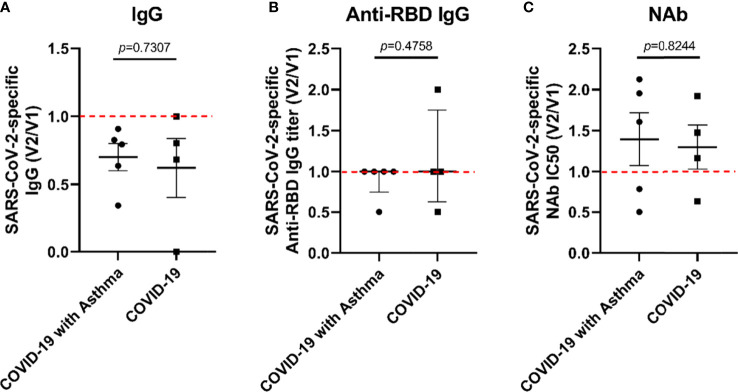
The decay rate of SARS-CoV-2-specific antibodies in COVID-19 survivors with/without asthma. The rate of SARS-CoV-2-specific antibody decay was expressed as the ratio of the antibody value at the second follow-up (V2) to the antibody value at the first follow-up (V1). The decay rate of SARS-CoV-2-specific IgG **(A)**, anti-RBD IgG titer **(B)**, and IC50 of NAb **(C)** were compared between COVID-19 with asthma and COVID-19 subjects. Unpaired t-tests were performed for comparison between two groups.

At 16 months post-discharge, a second follow-up was conducted on the COVID-9 survivors. At this stage, COVID-19 survivors who received SARS-CoV-2 vaccines are excluded. It was found that the decay rate of SARS-CoV-2-specific Nabs, anti-RBD IgG, and total IgG in the COVID-19 with asthma group (n=5) was not significantly different from those of the COVID-19 without asthma group (n=4) (**Figures 4A–C**).

In light of these results, we analyzed the percentages of circulating Tfh (cTfh) cells and SARS-CoV-2-specific memory B cells (MBCs) in the peripheral blood of 34 subjects based on sample availability. This was conducted in the COVID-19 with asthma (n=6), COVID-19 with allergy/allergies (n=6), COVID-19 only (n=13), asthma (n=6) and the healthy control group (n=3). The median percentage of cTfh cells in the COVID-19 with asthma group was higher than that observed in the other four groups, but there were no statistical differences (p=0.1296, **Figure 3E**). There were also no significant differences in the frequencies and ratios of cTfh1/cTfh2 cells between the five groups (**Figures 3F, G, I**), while the proportion of cTfh17 cells in the COVID-19 group was lower than in the asthma group (p<0.05, **Figure 3H**). The frequencies of the B cell subset, and MBCs, were not different between the five groups (**Figures 3J, K**). The median percentage of SARS-CoV-2-specific MBCs in COVID-19 survivors was higher (but not statistically significant) than that in asthma patients and healthy controls (overall p=0.084), while there were no significant differences between the COVID-19 patients with, and without, asthma (**Figure 3L**).

### Correlation Analyses

To explore the relationships between SARS-CoV-2-specific T cell responses and memory humoral immunity with Th2 inflammation, immune imbalance, and percentages of cTfh cells in COVID-19 survivors at the 8-month follow-up, further correlation analysis was conducted.

**Figure 5:**
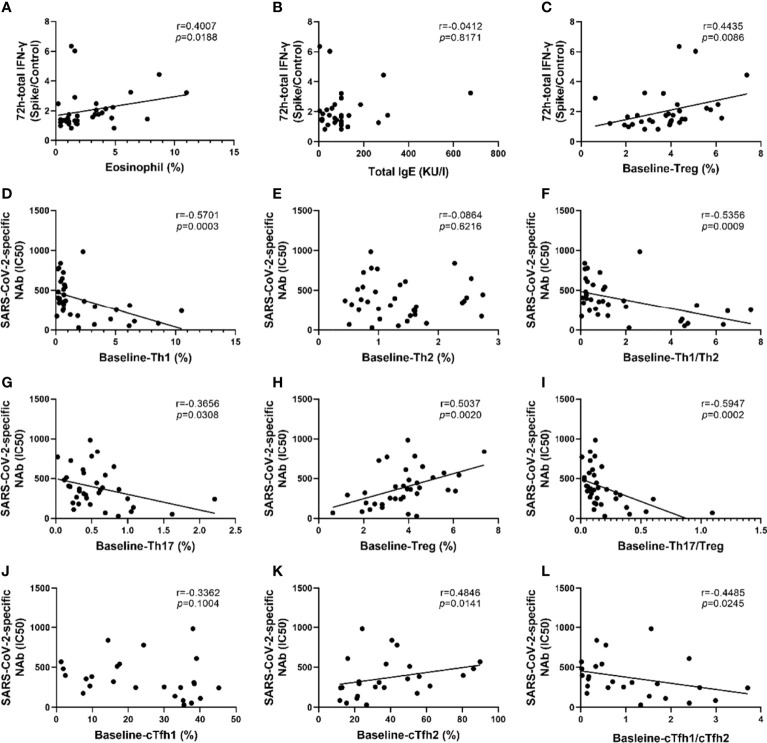
SARS-CoV-2-specific memory T cell responses and neutralizing antibodies were associated with T lymphocyte subsets and eosinophil proportions. **(A–C)** Correlation analysis of SARS-CoV-2 memory T cell response with eosinophils proportion, total IgE, and the percentage of Treg cells. **(D–I)** Correlation analysis of SARS-CoV-2-specific NAbs with the proportions of Th1 cells, Th2 cells, Th17 cells, and Treg cells, the ratio of Th1/Th2 and Th17/Treg. **(J–L)** Correlation analysis of SARS-CoV-2 specific NAbs with the proportions of cTfh1 cells, cTfh2 cells, the ratio of cTfh1/cTfh2. Each dot represents an individual subject. The black solid line of linear regression represents the overall trend. R and *p* value are calculated using Spearman’s correlation.

As shown in **Figure 5**, the strength of the SARS-CoV-2-specific T cell response was directly proportional to the percentages of eosinophils and Treg cells (p=0.0188 and p=0.0086, respectively, **Figures 5A, C**). However, no significant correlation was found between IgE and the SARS-CoV-2-specific T cell response (**Figure 5B**). Additionally, no significant correlation was found between SARS-CoV-2-specific T cell responses and lymphocyte subsets(**Figures E5A–E**).

Intriguingly, the results of the correlational analysis showed that the IC50 values of all COVID-19 survivors were negatively correlated with Th1 cells and the Th1/Th2 ratio, with R values of -0.5701 and -0.5356, respectively, and P values of 0.0003 and 0.0009, respectively (**Figures 5D, F**). The IC50 value was positively correlated with the proportion of Treg cells (r=0.5037, p=0.002) whereas it was negatively correlated with the proportion of Th17 cells (r=-0.3656, p=0.0308) and Th17/Treg ratio (r=-0.5947, p=0.002) (**Figures 5G–I**). Moreover, the IC50 value was positively correlated with cTfh2% and negatively correlated with the ratio of cTfh1/cTfh2 (all p<0.05, **Figures 5K, L**). Although not statistically significant, there was a trend of negative correlation between cTfh1 cells and IC50 value (**Figure 5J**). There was no significant correlation between the IC50 value with the percentage of eosinophils or IgE (**Figures E5F, H**).

## Discussion

In this study, we described and compared the differences in clinical characteristics, lymphocyte subsets, and levels of SARS-CoV-2-specific cellular and humoral immunity among COVID-19 survivors with and without asthma. Our research results suggest that the survivors with asthmatic comorbidity had higher levels of SARS-CoV-2-specific NAbs after eight months of recovery from COVID-19, and that the level of NAbs was related to the patient’s basic immune Th2/Th1 polarization status. Nevertheless, there were no significant differences in SARS-CoV-2-specific cellular immunity levels between patients 8 months after recovery from COVID-19 with and without asthma.

Eight months after discharge, some COVID-19 survivors still had persistent symptoms, abnormal lung diffusion function, and abnormal CT scores. These results are consistent with those of a previous longitudinal follow-up study (27). However, the incidence of these abnormalities in the present study did not differ significantly between COVID-19 survivors with or without asthma comorbidity. The pulmonary ventilation function of the COVID-19 survivors with asthma comorbidity was lower than that of those without asthma, which may be related to asthma itself.

The specific memory immunity of SARS-CoV-2 in COVID-19 survivors is closely related to the risk of SARS-CoV-2 reinfection and the outcome of COVID-19 (28, 29). However, little information is about the memory immune of SARS-CoV-2 in COVID-19 survivors with asthma. Our findings suggest that SARS-CoV-2-specific memory T cells are still present in the majority of COVID-19 survivors 8 months after discharge, which is similar to previous studies (30, 31). However, there was no significant difference in the levels of SARS-CoV-2-specific T cell responses between COVID-19 survivors with asthma and those without asthma. Furthermore, we found that the levels of SARS-CoV-2-specific T cell memory responses were positively correlated with eosinophil percentages in 34 COVID-19 survivors at 8 months post-discharge. Eosinophils play an important role in asthma and allergic diseases and have a potential role in promoting virus clearance and antiviral host defense (32, 33). Previous observations have demonstrated that eosinophils respond to airway viruses. Influenza A virus peptide stimulated eosinophils can induce T cell activation and promote host cellular immunity, which is consistent with our results (33, 34). The proportion of eosinophils in peripheral blood from severely affected COVID-19 patients is reduced, which in turn is related to the progression and prognosis of severe COVID-19 patients (35–37). A retrospective study reported by Ferastraoaru et al. concluded that increased eosinophils in asthmatic patients during hospitalization were associated with reduced mortality from COVID-19 (38). COVID-19 survivors with elevated eosinophils may therefore have a reduced risk of severe reinfection with SARS-CoV-2.

Th2/Th1 imbalance may affect patients’ susceptibility to SARS-CoV-2 and clinical outcomes in SARS-CoV-2 infection (39, 40). Th2 cytokines can down-regulate the expression of airway epithelial angiotensin-converting enzyme 2 (ACE2) (21, 41, 42), which is the primary receptor of SARS-CoV-2, thereby reducing the prevalence and severity of COVID-19 patients. On the other hand, COPD inflammatory airway disease, which is typically Th1-skewed immunity, had an increased rate of COVID-19 infection and increased severity of the disease (20, 43). The majority of asthmatics demonstrated a predominantly Th2 immune response (44, 45). Our study explored the basic immune cell subsets of COVID-19 patients at the 8-month follow-up and found that the percentage of Th2 cells at baseline in COVID-19 patients with asthma was higher than in patients without asthma, and the Th1/Th2 ratio was lower than in patients without asthma. It is worth noting that although previous studies have shown that Th1 type cellular immunity plays a major role in the body’s fight against SARS-COV-2 infection (46, 47), in our data, there is no correlation between Th2/Th1 imbalance and SARS-CoV-2-specific cellular immunity in COVID-19 patients during recovery.

At 8 months post-discharge, the level of NAbs in the COVID-19 survivors with asthma was higher than for those without asthma. NAbs play an important role in preventing SARS-CoV-2 infection, and the level of NAbs is positively correlated with the severity of this disease (48, 49). Consistent with previously published literature, the present research found that the IC50 value in severe COVID-19 convalescent patients was higher than that in non-severe patients at the 8-month follow-up stage (50) (**Figure 3D**). COVID-19 survivors with asthma had a lower rate of severe COVID-19 during hospitalization, but a higher level of SARS-CoV-2-specific NAbs eight months after discharge, which strengthens the evidence that COVID-19 survivors with asthma had a higher level of specific humoral immunity than that without asthma. What’s more, there was no significant difference in the NAbs decay rates between COVID-19 survivors with asthma and those without asthma.

We carried out further research to discover the reasons behind this phenomenon indicating higher levels of NAbs in COVID-19 survivors with asthma. There was no significant difference in the levels of SARS-CoV-2 specific MBCs between the COVID-19 survivors with asthma and those without asthma at the 8-month follow-up. Moreover, we found that the value of IC50 was directly proportional to the percentage of cTfh2 cells and inversely proportional to the ratio of cTfh1/cTfh2 cells. No positive association was found between NAbs and the proportion of cTfh1 cells, unlike previous findings regarding COVID-19 survivors at the one month of follow-up (24). The cTfh cells are a unique subset of CD4^+^T cells, whose main role is to help B cells establish germinal center responses and produce high-affinity antibodies (51, 52). Our study depicts a close association between the ratios of cTfh1/cTfh2 cells with the SARS-CoV-2-specific antibody production in COVID-19 survivors.

In addition, the values of IC50 in COVID-19 convalescent individuals was positively correlated with the percentage of Treg cells and negatively correlated with the percentage of Th1 and Th17 cells, Th1/Th2, and Th17/Treg ratios. These results seem to be consistent with other research which found Ab-negative recovered COVID-19 individuals had higher Th17 cells percentages, and higher Th1/Th2 and Th17/Treg ratios, compared with Ab-positive individuals (53). Previous studies on immunity to infection with SARS-CoV-2 have shown that CD4^+^ T cell responses are mainly polarized to the Th1 type (54). Th2 polarized immunity can not only stimulate antibody production but also suppress Th1 cell-mediated immunity (55, 56). This might explain why the Th1/Th2 ratio was inversely correlated with neutralizing antibody levels. Increased Th17 response and disturbance ratios of Treg/Th17 may be contributed to the excessive inflammation of COVID-19 (57, 58). COVID-19 survivors with Th17-polarized immune responses and Th17/Treg dysfunction have lower levels of specific humoral immunity, possibly partly at the expense of Th2 humoral immunity (59). Our results confirmed that the Th17/Treg immune balance plays an important role in the specific immunity of SARS-CoV-2 infection.

Treg cells are a class of CD4^+^T cells, which play a critical role in maintaining immune homeostasis (60, 61). In our study, Treg cells exhibited a strong positive correlation with the strength of cellular and humoral memory immune response to SARS-CoV-2. Treg cells play an important role in the pathogenesis of COVID-19 by inhibiting adaptive immune responses. In addition, the percentage of Treg cells in patients with severe COVID-19 showed a decreasing trend (62). COVID-19 patients exhibit a heavily hyperinflammatory milieu during the active phase of the disease, accompanied by high consumption of circulating Treg cells, so we speculate that Treg cells in recovered donors show a feedback increase during the convalescence stage. Meanwhile, severe COVID-19 patients have stronger SARS-CoV-2-specific immune response during recovery (63, 64), which may partly explain the positive correlation between the proportion of Treg cells and SARS-CoV-2-specific T cell immunity and NAbs in COVID-19 patients after 8 months of recovery. However, the specific mechanism of action of Treg cells in SARS-COV-2 immune memory needs to be further explored.

ICS, alone or combination with bronchodilators, are used extensively in the treatment of asthma and affect human immunity. Inhaled or systemic can inhibit the production of the critical antiviral mediators’ Type I and III interferons (65). *In vitro* studies have suggested that corticosteroids may impair antiviral innate immune responses (66, 67). A result of a previous study indicated that in mice models of allergic asthma, inhaled glucocorticoids prevented the response of CD8^+^T cells (68). However, as corticosteroids suppress type 2 inflammation, their use in COVID-19 with asthmatic patients may thus lead to a beneficial effect (69). Furthermore, in patients with immune deficiency diseases, glucocorticoids may affect the activation of B cells through various pathways, thereby reducing the production of antibodies and impairing humoral immunity (70, 71). In our study, 6 out of 11 (55%) asthma patients who recovered from COVID-19 were treated with ICS/LABA. There were no significant differences in the SARS-CoV-2-specific cellular and humoral immune memory between COVID-19 survivors treated with and without ICS/LABA (**Figure E7**).

The present study has several limitations. Firstly, our cohort is of relatively small size, because there are fewer COVID-19 patients with asthma that can be recruited. A larger cohort is needed to corroborate this issue. Secondly, this research was restricted by analyzing SARS-CoV-2-specific cells to those recognizing the spike protein, which elicits a limited CD4^+^ or CD8^+^T cell response. Besides this, we did not perform CXCR5^-^CD45RA^-^/CD45RO^+^ gating strategies to rigorously definite effector T cells, which should be improved in the future study. Thirdly, our experimental methods for detecting SARS-CoV-2 specific T cells and memory B cells are not sufficiently comprehensive. The ELISpot method can supplement the evaluation of the cellular immune memory. Humoral memory responses can be assessed by analyzing changes in the proportion of MBC and changes in the level of neutralizing antibodies released by MBC after antigenic peptide stimulation. Last, our results were applied to adult patients and mostly Th2-high asthma. Therefore, pediatric asthma or Th2-low asthma within COVID-19 patients may behave differently with regards to immune intensity and duration thereof.

The level of NAbs in COVID-19 survivors with asthma was higher than for those without asthma at 8 months follow-up. In the future, further investigation will help improve our understanding of the interaction between immune polarization and immune protection against SARS-CoV-2.

## Data Availability Statement

The original contributions presented in the study are included in the article/**Supplementary Material**. Further inquiries can be directed to the corresponding authors.

## Ethics Statement

The studies involving human participants were reviewed and approved by Ethics Committee of Tongji Hospital, Tongji Medical College, Huazhong University of Science and Technology. The patients/participants provided their written informed consent to participate in this study.

## Author Contributions

MX, RG, JY and RZ conceived the study and planned the experiments. JY, LC, BW and ML carried out the experiments. JY, WB, SZ, LQ, CZ, SX, QJ, LY, QX, and RZ supported the clinical aspects of the study. JY, LC, RG and MX analyzed the data. MX, RG, JY and LC participated in data interpretation and scientific discussion. JY and MX drafted and revised the manuscript. All the authors reviewed and approved the final version of the manuscript. The corresponding author attests that all listed authors meet authorship criteria and that no others meeting the criteria have been omitted.

## Funding

This research was jointly funded by the CAS-VPST Silk Road Science Fund 2021 (GJHZ2021134), the National Natural Science Foundation of China (32170949) and the Natural Science Foundation of Hubei Province of China (2019CFA076).

## Conflict of Interest

The authors declare that the research was conducted in the absence of any commercial or financial relationships that could be construed as a potential conflict of interest.

## Publisher’s Note

All claims expressed in this article are solely those of the authors and do not necessarily represent those of their affiliated organizations, or those of the publisher, the editors and the reviewers. Any product that may be evaluated in this article, or claim that may be made by its manufacturer, is not guaranteed or endorsed by the publisher.

## Abbreviations

COVID-19, Coronavirus disease 2019; SARS-CoV-2, Acute respiratory syndrome coronavirus 2; GINA, Guidance within the Global Initiative for Asthma; NAb, Neutralizing antibody; IC50, Half maximal inhibitory concentration; ACE2, Angiotensin-converting enzyme 2; FEV1, Forced expiratory volume in one second; FVC, Forced vital capacity; MVV, Maximum voluntary ventilation; MEF, Maximal expiratory flow at 50% of FVC; PD20, Provocation dosage; 6MWD, 6-min walk distance; ACT, Asthma control test; S, Spike; RBD, Receptor-binding domain; Th cell, Helper T cell; Tfh cell, Follicular helper T cell; Treg cell, Regulatory T cell; MBC, Memory B cell.
