# Supplementary material for: SARS-CoV-2-Specific Adaptive Immunity in COVID-19 Survivors With Asthma
Source: Front Immunol. 2022 Jul 18;13:947724. doi: 10.3389/fimmu.2022.947724 (PMC9339657; doi:10.3389/fimmu.2022.947724)
Supplement: Supplementary file 1 [file DataSheet_1.docx]

**Supplementary Materials**

**Methods**

**Study subjects**

In total, 36 convalescing COVID-19 individuals (who also had asthma n=11, allergies n=8, or COVID-19 only n=17) were recruited. The patients had received positive laboratory test results using the SARS-CoV-2 nucleic acid test between January 2020 to March 2020, in Wuhan, China. The diagnosis of severe COVID-19 was based on the National Health Commission of the People’s Republic of China’s interim guidance (1). Asthma diagnosis was based on the guidance within the Global Initiative for Asthma 2020 (GINA 2020) (2), and was confirmed by at least one of the following key criteria: bronchoprovocation test with methacholine PD20 < 2.505 mg or a bronchodilation FEV1 change > 200 mL and 12%. Type 2-high asthma was defined as meeting one of the following characteristics: total IgE > 100 kU/L, fractional exhaled nitric oxide > 30 ppb, blood eosinophil > 250 cells/μL, atopy or comorbidity with an allergic disease (3). All allergic subjects were diagnosed with allergic symptom and had at least one specific IgE (≥0.35 kUI/L) toward common allergens performed at follow-up (4).

The COVID-19 survivors without asthma and the COVID-19 survivors with allergies were included in this study after being matched by age and sex to the COVID-19 survivors with asthma. Patients with a history of other chronic respiratory disease and those who had been identified with a respiratory infection within the previous two weeks were excluded from the study. Similarly, age- and sex-matched asthmatic patients (n=10) and healthy donors (n=9) who were not vaccinated with COVID-19 vaccine were included, these control group patients tested negative for SARS-CoV-2 using the nucleic acid test. Asthmatic patients who were in an acute stage of asthma were excluded. Healthy controls who had no atopy, chronic respiratory diseases, systemic infections, autoimmune disorders, or cancers were all considered appropriate for inclusion.

The COVID-19 patients were followed up at about 8 months (visit 1) and 16 months (visit 2) after discharge (Table E1). The patients were interviewed and underwent clinical questionnaires and physical examinations. Data were also collected regarding demographic information, clinical characteristics (including comorbidities, smoking history, and atopy history), laboratory outcomes (routine blood test and SARS-CoV-2-specific antibodies test), chest imaging findings, pulmonary function, standardized 6-min walk distance (6MWD) test, and treatment details. For asthmatic patients, with or without COVID-19, clinical characteristics (including their Asthma Control Test (ACT) scores and details of maintenance treatments for their asthma) were also collected. All of the clinical data were checked by two physicians. The 11 COVID-19 survivors with asthmatic comorbidities were also reported upon the clinical characteristics in a previous study by our research group (unpublished data).

All participants gave written informed consent before being included. The present study was approved by the Ethics Committee of Tongji Hospital, Tongji Medical College, Huazhong University of Science and Technology (IRB ID: TJ-IRB20210115). The study complied with all of the relevant ethical regulations including The Code of Ethics of the World Medical Association (Declaration of Helsinki).

**PBMC isolation and SARS-CoV-2 Spike peptide pools stimulation**

Whole blood was collected into an EDTA-K2 anticoagulant tube. Peripheral blood mononuclear cells (PBMCs) were isolated using density-gradient centrifugation according to the manufacturer’s instructions (Ficoll-Paque Plus, USA). The plasma samples were stored at -80°C in multiple aliquots. Isolated PBMCs were cryopreserved in cell freezing medium (Recovery Cell Culture Freezing Medium, Gibco, USA), and stored in liquid nitrogen for later experiments.

Resuscitated PBMCs with > 80% viability were cultured in RPMI 1640 medium (Gibco, USA) supplemented with 10% heat inactivated fatal bovine serum (FBS, Gibco, USA). Overnight rested PBMCs were then plated at 1×10^6^ cells/well and stimulated with the SARS-CoV-2 Spike peptide pools (11aa overlapping 15 mer peptides from Genscript, China) at 1.6 ug/mL/peptide and cultured for 24 hours or 72 hours (37°C, 5% CO_2_). Phosphate-buffered saline (PBS) and phorbol 12-myristate 13-acetate (PMA,25 ng/mL) plus ionomycin (500 ng/mL) were used as negative and positive controls, respectively. In order to conduct intracellular cytokine staining (ICS), PBMCs were cultured in the presence of SARS-CoV-2-specific spike peptide pools for 24 h at 37°C and brefeldin A (BFA, 10 µg/mL) was added 6 h into the culturing protocol. Unstimulated PBMCs after thawing was defined as baseline and ICS flow cytometry was also performed.

**Flow cytometry**

Prior to labeling the cells with antibodies, the cells were blocked with Human TruStain FcX (Biolegend) for 10 minutes. Surface-markers, including CD3, CD4, CD8, CD25, CD127, CD183, CD196 and CD294 were used for staining the cells. After surface staining, the cells were fixed and permeabilized in accordance with the manufacturer’s instructions, and were then stained with IFN-γ, TNF-α, IL-4, and IL-10.

The surface markers used in the Tfh cell flow cytometry analysis were CD3, CD4, CXCR5, PD-1, CD183, and CD196. The surface markers used for SARS-CoV-2-specific memory B cell analysis were CD19, CD10, CD27, CD38, IgD, PE-RBD (SARS-CoV-2-spike protein receptor-binding domain) and PE/Cy7-RBD. These final two panels also included a Zombie viability stain. Surface markers and intracellular cytokine staining markers are listed in detail in Table E2.

Negative controls including PBS were run for each sample. Isotype controls were used to correct for nonspecific binding, and a fluorescence minus one (FMO) control was used to determine positive cells. All samples‘ analyses were conducted on a BD LSRFortessa (BD Biosciences, USA) flow cytometer. The gating strategy followed (Figure E1) was used to analyze the immunophenotypes of lymphocytes.

**Measuring lymphocyte proliferation in vitro using CFSE-like fluorescent dyes**

Carboxyfluorescein diacetate succinimidyl ester (CFSE; Biolegend, USA) fluorescent dyes were dissolved in DMSO as 5 mM stock solutions (stored at −20℃). Thawed PBMCs were resuspended in PBS/2%FBS at a final concentration of 1×10^6^ cells/mL, 5mM CFSE (final 2.5μM) was added into each tube containing the cells, which were then incubated for 10 min at 37°C. Staining was quenched using a 5X volume of RPMI1640/10%FBS, which was incubated on ice for 5min. The cells were then washed three times in the culture medium. Cells were cultured at 37°C in 5% CO_2_ alongside the PBS control stimulation sample and the SARS-CoV-2 spike peptide pools for 72 hours, thereafter antibody staining and analysis by flow cytometry were conducted.

**IFN-γ secretion assays**

After the PBMCs had been stimulated by the PBS control or the SARS-CoV-2 spike peptide pools for 72h, the culture supernatant was harvested for detection of human IFN-γ via ELISA (ELISA MAX^TM^ standard Set Human IFN-γ, Biolegend, USA). The detection limit of the IFN-γ assay kit is 7.8 pg/mL. Dilution factors of the samples were determined during a preliminary experiment.

The 96-well clear plates were coated with IFN-γ monoclonal antibody overnight at 4°C. The next day the plates were blocked with 100 μl assay diluent at room temperature for 1h. After washing with PBST buffer (0.1% Tween 20 in PBS), the diluted standards and samples were added to the plates and incubated for 2h at room temperature. Then, the ELISA plates were incubated with IFN-γ detection antibodies for 2h at room temperature. Following four washes with PBST buffer, the plates were incubated with diluted avidin-HRP solution at room temperature for 30 min. After washing, TMB substrate solution (Biolegend, USA) was added to each well and the plates were incubated in the dark for 20 minutes. The reactions were stopped with a stop solution. Plates were read at 450 nm, and ODs had the background subtracted.

**ELISA analysis of SARS-CoV-2 antigen-specific plasma antibody**

The RBD protein derived from the SARS-CoV-2 isolate Wuhan-Hu-1 (GenBank MN908947.3) was used as a coating antigen. ELISA plates were coated with 50μL of 4μg/mL protein in PBS overnight at 4°C and blocked with blocking buffer (PBS containing 3% skimmed milk) at 37°C for 1h. The plates were washed with PBST (PBS containing 0.05% Tween-20), then serially diluted plasma samples were added and incubated at 37°C for 1.5 h. HRP-conjugated goat anti-human IgG (Fab specific) antibody (1:5000) was used as a secondary antibody for detection. Binding was measured with the subsequent addition of substrate diammonium 2,2-azinobis (3-ethylbenzothiazoline-6-sulfonate) (ABTS; Life technology, USA), and the absorbance signal was read at 405 nm.

**SARS-CoV-2 pseudotyped virus neutralization assay**

SARS-CoV-2 pseudotyped viruses were generated as previously described (5, 6). In brief, 293T cells were transfected with pCAGGS-SARS-CoV-spike△18 (We've truncated 18 amino acids from the C-terminal) and cultured at 37°C in 5% CO_2_ for 24 hours. Next, the medium was removed and VSV-△G-EGFP (The missing G protein was replaced with EGFP)was added to infect the cells for 2 hours, after that the cells were washed with PBS three times. DMEM supplemented with 2% FBS (Gibco, USA) was added and the supernatants were collected the following day and filtered using a 0.45-μm filter.

Three fold diluted plasma was incubated with the SARS-CoV-2 pseudovirus at 37°C for 1 hour. This mixture was added to a monolayer of Vero-E6 cells in a 96-well plate and incubated for 24 hours. Luminescence was measured using an Operetta high-content imaging system combined with Harmony imaging and analysis software (PerkinElmer). We set up multiple holes for each experiment. The inhibition rate of each dilution was evaluated and the half maximal inhibitory concentration (IC50) was calculated using nonlinear regression in SPSS.

**Statistical analysis**

Categorical variables were described as frequency rates and percentages. Continuous variables were described using the mean ± SD or the median and interquartile range (IQR) values. Student’s t-test and Mann Whitney U test were performed for comparing continuous variables where only two groups were being compared. The X^2^ test and Fisher exact test were used to analyze categorical variables as appropriate. Ordinary one-way analysis of variance (ANOVA, for normally distributed data) or a Kruskal-Wallis test (for non-normally distributed data) were utilized to compare multiple groups. Tukey (for normally distributed data) or Dunn’s multiple comparison (for non-normally distributed data) were used to compare between these groups. All of the statistical tests used were two-sided. The flow cytometry data were analyzed with FlowJo V10. SPSS software V.25.0 and GraphPad Prism 8 were used for plotting graphical information and statistical analysis.


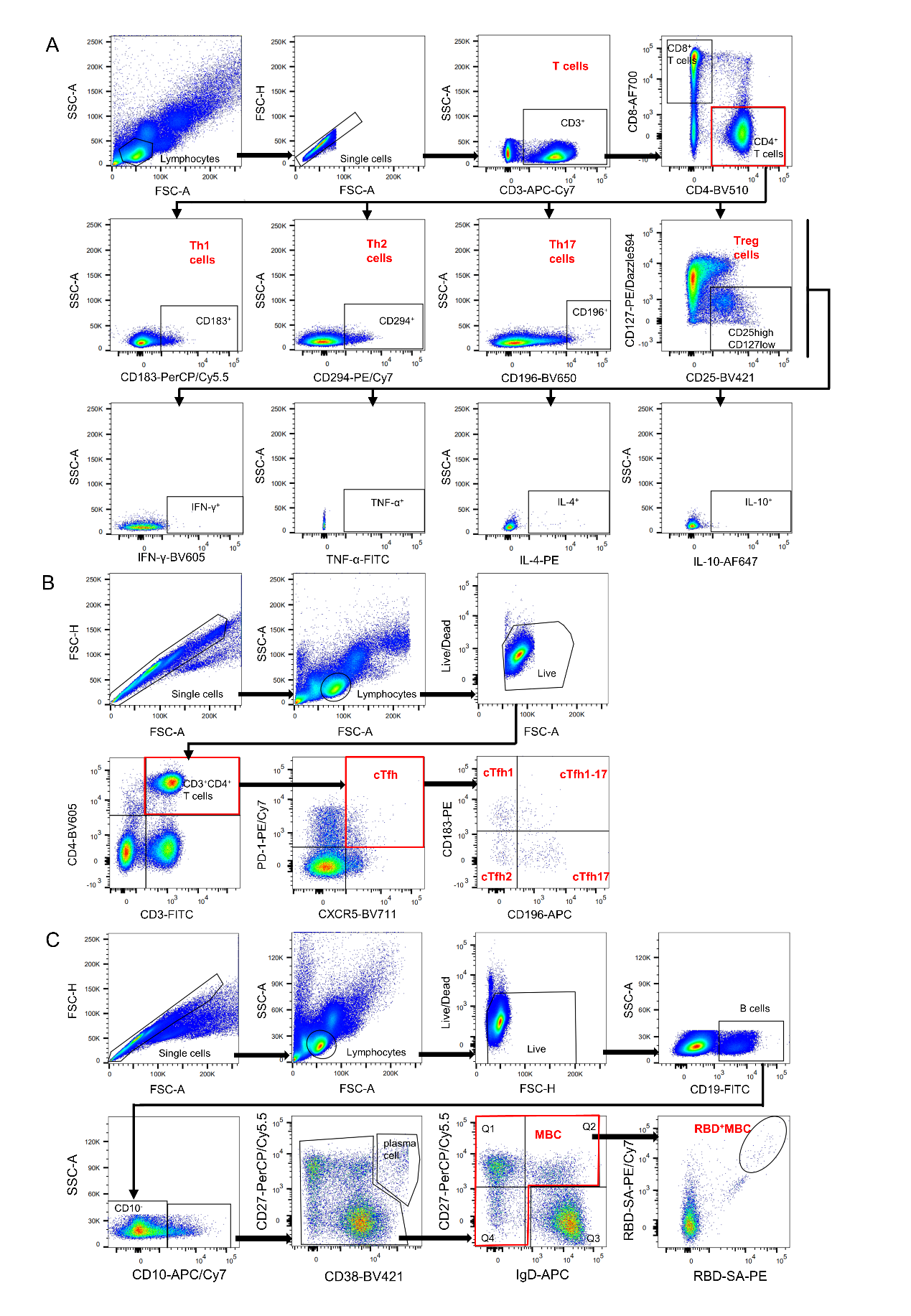


**Figure E1.** **Gating strategy for lymphocyte subsets, intracellular cytokine, cTfh cells and B cells.** Example of flow cytometry gating strategy. (A) Mononuclear cells were gated out of total events followed by subsequent singlet gating. CD3^+^T cells were then divided as CD4^+^ or CD8^+^ T cells. CD4^+^T cells then divided into four subsets of lymphocytes, CD183^+^T cells (Th1), CD294^+^T cells (Th2), CD196^+^T cells (Th17) and CD25^high^CD127^low^T cells (Treg). The next gating strategy is to analyze the percentage of IFN-γ^+^, TNF-α^+^, IL-4^+^ and IL-10^+^ in different lymphocyte subsets. (B) Gating strategies on different peripheral circulating CD4^+^T cell subsets, including CXCR5^+^PD-1^+^ cTfh cells, CD183^+^CD196^–^cTfh cells (cTfh1), CD183^–^CD196^–^cTfh cells (cTfh2), and CD183^–^CD196^+^ cTfh cells (cTfh17). (C) Gating strategy used to identify SARS-CoV-2-Spike RBD specific B cells. Fluorescence minus one (FMO) and isotype controls were used as a guide to differentiate positive and negative populations.


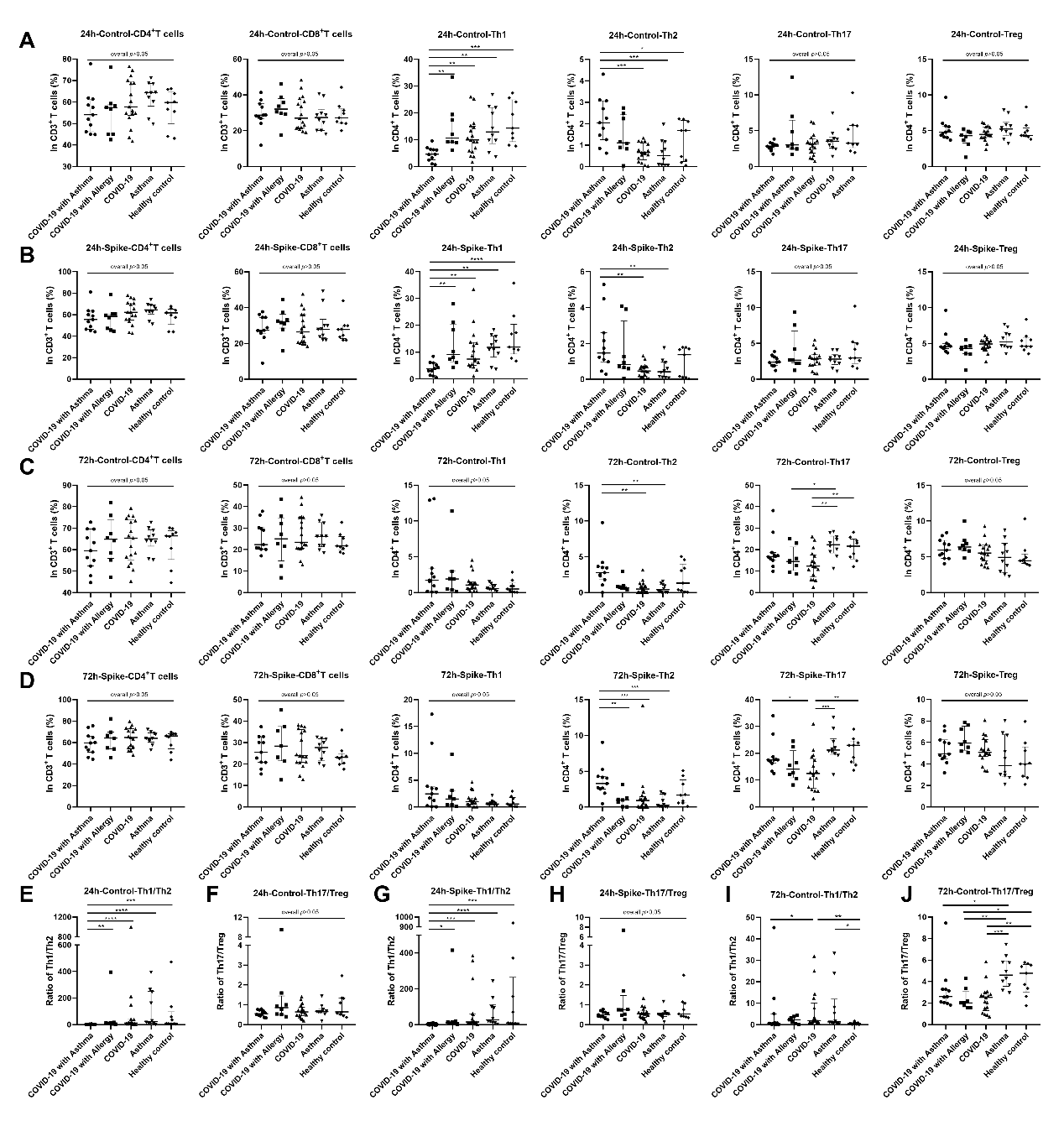


**Figure E2. Blood lymphocyte subsets at 24 hours and 72 hours after SARS-CoV-2 Spike peptide pools stimulation among COVID-19 groups with/without asthma or allergy.**

Analysis of blood lymphocyte subsets in COVID-19 with asthma group (n=11), COVID-19 with allergy group (n=8), COVID-19 group (n=17), asthma group (n=10) and healthy control group (n=9).

(A, B, E-H) Lymphocyte subsets were detected by flow cytometry for CD4^+^T cells, CD8^+^T cells, Th1 cells, Th2 cells, Th17 cells, and Treg cells at 24 hours after stimulation by SARS-CoV-2 Spike peptide pools or PBS control. (C, D, I, J) Lymphocyte subsets were also detected at 72 hours after stimulation by SARS-CoV-2 Spike peptide pools or PBS control. The percentages of and the ratios of CD4^+^/CD8^+^, Th1/ Th1, and Th17/Treg in the five groups were analyzed. Data were expressed as median± interquartile range (IQR) and tested using the Kruskal-Wallis test and Dunn’s test. ^*^ p<0.05, ^**^ p<0.01, ^***^ p<0.001, ^****^ p<0.0001; ns, not significant.


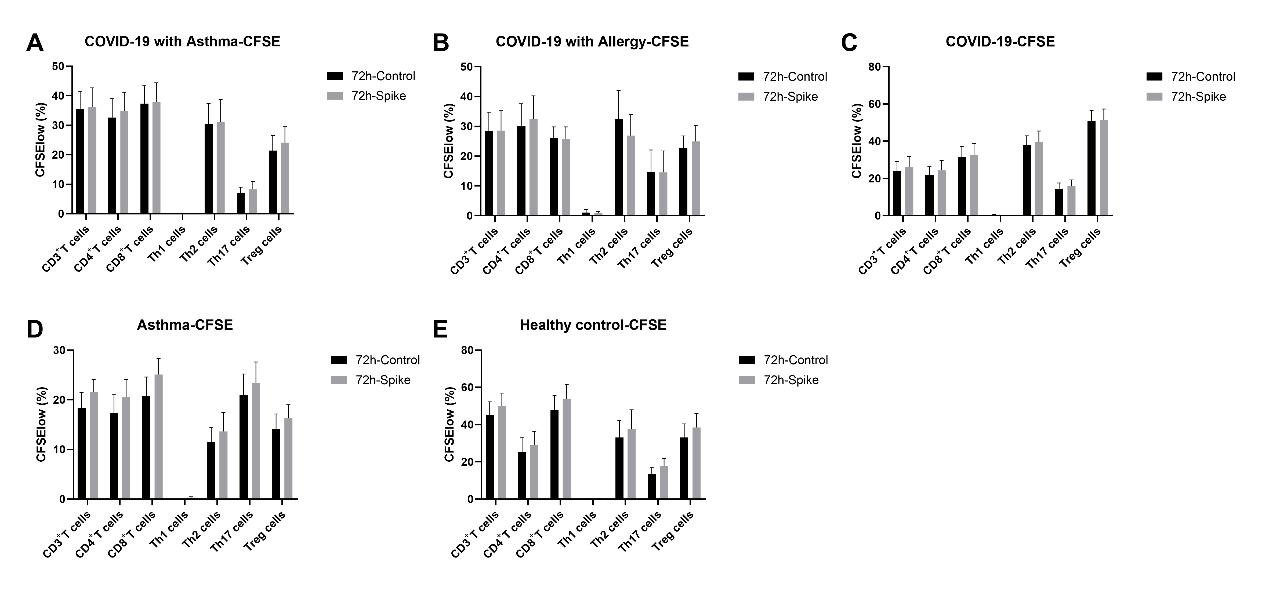


**Figure E3. Lymphocyte proliferation at 72 hours by stimulation of SARS-CoV-2 Spike peptide pools**

The proliferation of lymphocyte subsets (CD3^+^T cells, CD4^+^T cells, CD8^+^T cells, Th1 cells, Th2 cells, Treg cells, and Th17 cells) among the five groups were detected by CFSE method at 72 hours by stimulation of SARS-CoV-2 Spike peptide pools or PBS control(A-E). The longitudinal axis in the figure shows the percentage of CFSE low cells of the lymphocyte subsets, and bars represent SEM. SEM, Standard Error of Mean.


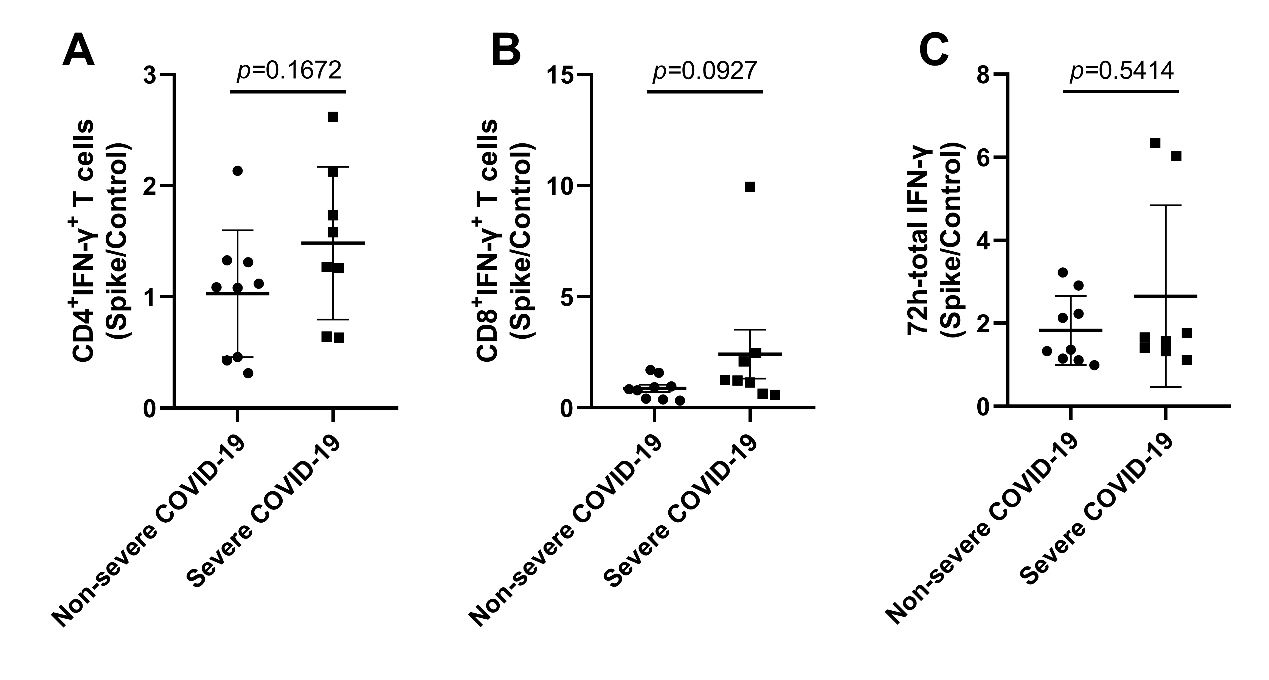


**Figure E4.** **SARS-CoV-2-specific T cell responses in severe and non-severe COVID-19 survivors.**

The ratio of specific T cell response by spike peptide pools stimulation compared with PBS control. (A, B) SARS-CoV-2-specific responses of CD4^+^ and CD8^+^ T cells in severe and non-severe COVID-19 survivors. (C) SARS-CoV-2-specific responses of total T cells in severe and non-severe COVID-19 survivors. Each point on the graph represents a single donor. Mann-Whitney tests were performed for comparison between two groups.


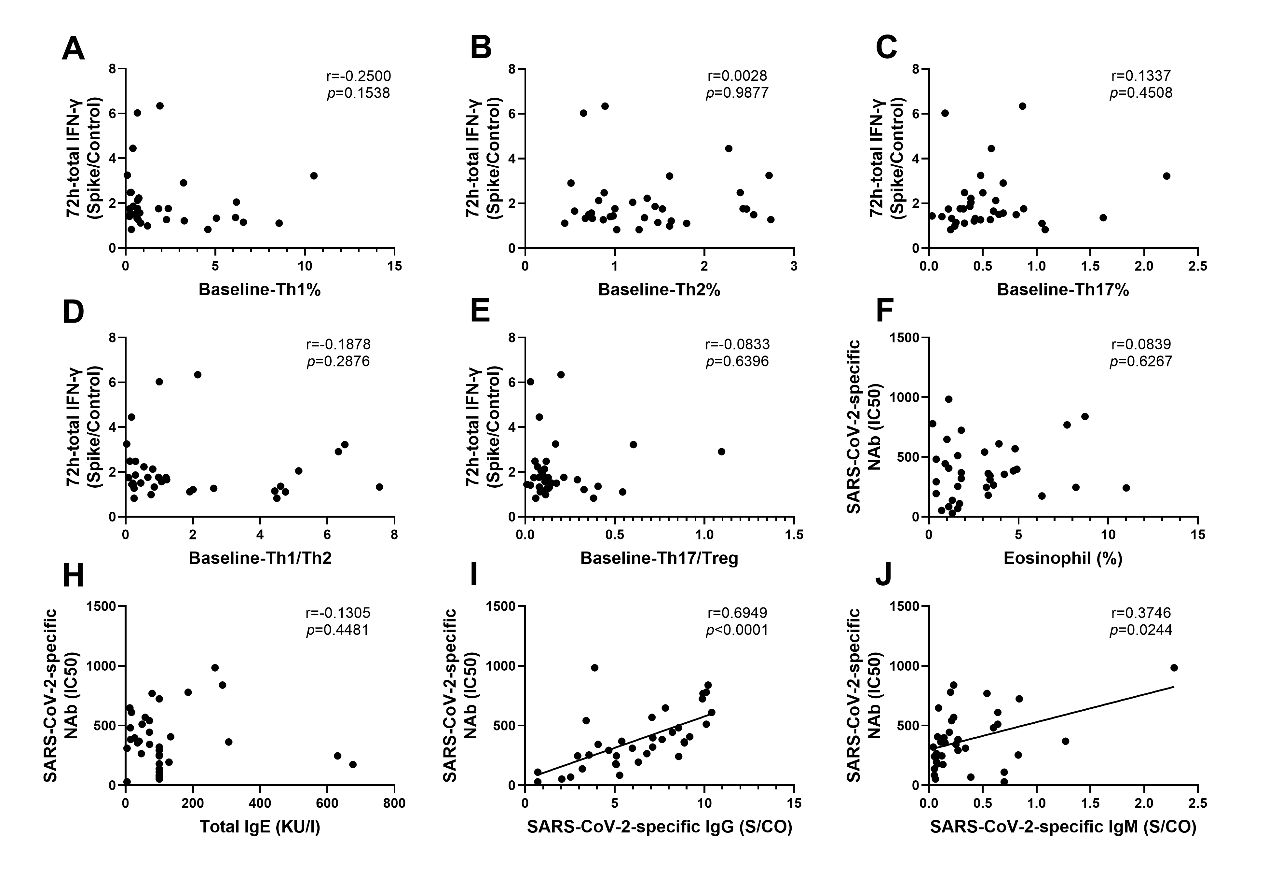


**Figure E5. Correlation analysis of SARS CoV-2-specific antibodies with T cell subsets**

(A-E) Correlation analysis of SARS-CoV-2 memory T cell response with the proportions of Th1 cells, Th2 cells, and Th17 cells, ratios of Th1/Th2 and Th17/Treg. (F, H) Correlation analysis of SARS-CoV-2-specific Nabs with eosinophils proportion and total IgE. (I, J) Correlation analysis of SARS-CoV-2-specific IgG and IgM with SARS-CoV-2-specific Nabs in COVID-19 survivors 8 months after recovery. Each dot represents an individual subject. The black solid line of linear regression represents the overall trend. R and *p* values are calculated using Spearman’s correlation.


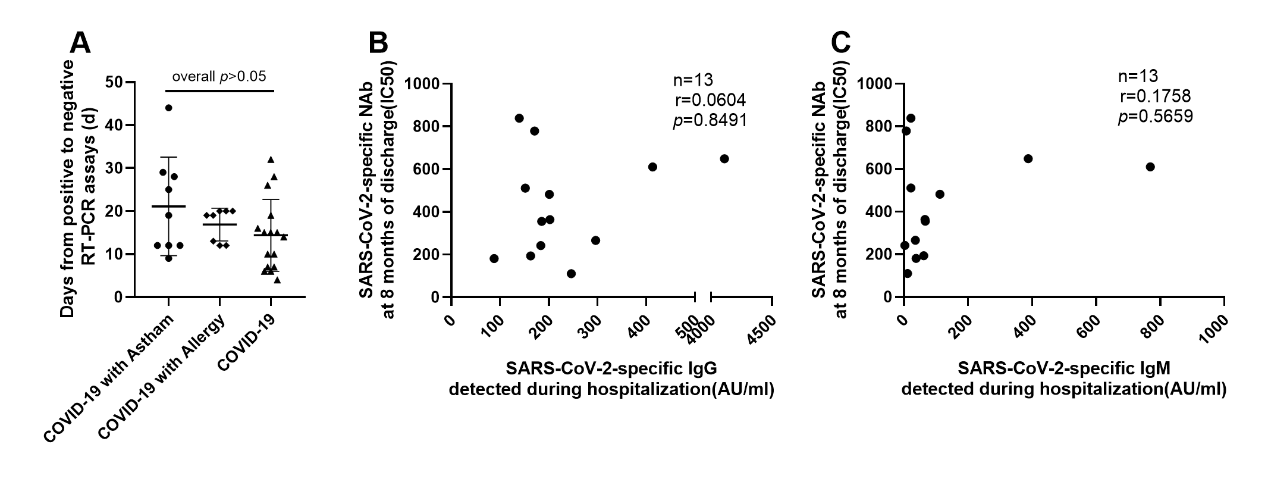


**Figure E6. Shedding time of SARS-CoV-2 and the correlations between SARS-CoV-2 specific antibodies during hospitalization and at follow-up.**

(A) Days of SARS-CoV-2 nucleic acid assays from the first positive to negative were compared in the COVID-19 with asthma group (n=9), COVID-19 with allergy group (n=8), and COVID-19 group (n=16). (B, C) Correlation analysis of the value of SARS-CoV-2-specific IgG and IgM detected during hospitalization and SARS-CoV-2-specific Nab in COVID-19 survivors 8 months after recovery. Each dot represents an individual subject. The black solid line of linear regression represents the overall trend. R and *p* values are calculated using Spearman’s correlation.


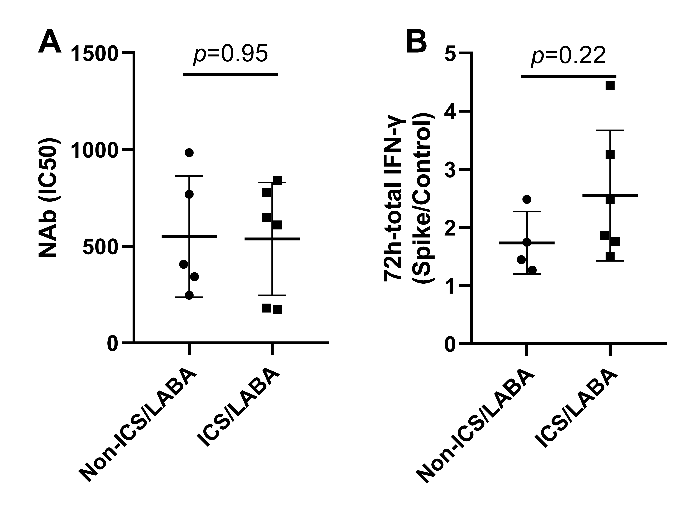


**Figure E7. SARS-CoV-2-specific neutralizing antibodies and memory T cell responses between COVID-19 survivors treated with and without ICS/LABA.**

The ratio of specific T cell response by spike peptide pools stimulation compared with PBS control. (A) The level of SARS-CoV-2-specific neutralizing antibodies between COVID-19 survivors treated with and without ICS/LABA. (B) SARS-CoV-2-specific responses of total T cells between COVID-19 survivors treated with and without ICS/LABA. Each point on the graph represents a single donor.

**Table E1. Comparative analysis of two follow-up times.**

|  | **Time** | **COVID-19**  **with Asthma** | **COVID-19**  **with Allergy** | **COVID-19** | ***p* value** |
| --- | --- | --- | --- | --- | --- |
| **Time from discharge to first follow-up, d** | Visit 1 | 239 ± 12 | 246 ± 15 | 245 ± 13 | 0.491 |
| **Time from discharge to second follow-up, d** | Visit 2 | 489.2 ± 8.17 | **-** | 482 ± 10.89 | 0.293 |

Data were expressed as mean ± SD. Comparisons were determined by one-way analysis of variance (ANOVA) test or Student’s t-test.

| **Reagents or Resource** | **Source** | **Identifier** | **Dilution** |
| --- | --- | --- | --- |
| **Antibodies utilized in the CD4^+^ and CD8^+^ T cell stimulated with Peptide** | | | |
| Anti-human CD3 APC (clone UCHT1) | Biolegend | Cat#300470 | 1:100 |
| Anti-human CD4 BV510 (clone SK3) | Biolegend | Cat#344634 | 1:100 |
| Anti-human CD8 AF700 (clone HIT8a) | Biolegend | Cat#300920 | 1:250 |
| Anti-human CD183 PerCP/Cy5.5 (clone G025H7) | Biolegend | Cat#353714 | 1:100 |
| Anti-human CD294 PE/Cy7 (clone BM16) | Biolegend | Cat#350118 | 1:100 |
| Anti-human CD196 BV650 (clone G034E3) | Biolegend | Cat#353426 | 1:100 |
| Anti-human CD25 BV421 (clone BC96) | Biolegend | Cat#302630 | 1:100 |
| Anti-human CD127 PE/Dazzle 594 (clone A019D5) | Biolegend | Cat#351336 | 1:100 |
| Anti-human IFN-γ BV605 (clone B27) | Biolegend | Cat#506542 | 1:100 |
| Anti-human TNF-α FITC (clone MAb11) | Biolegend | Cat#502906 | 1:100 |
| Anti-human IL-4 PE (clone MP4-25D2) | Biolegend | Cat#500810 | 1:100 |
| Anti-human IL-10 AF647 (clone JES3-9D7) | Biolegend | Cat#501412 | 1:100 |
| **Memory B cell cytometry panel** | | | |
| Anti-human CD19 FITC (clone HIB19) | BD Bioscience | Cat#555412 | 1:20 |
| Anti-human CD38 BV421 (clone HIT2) | BD Bioscience | Cat#562444 | 1:100 |
| Anti-human CD27 PerCP/Cy5.5 (clone M-T271) | Biolegend | Cat#356408 | 1:100 |
| Anti-human CD10 APC/Cy7 (clone HI10a) | Biolegend | Cat#312212 | 1:100 |
| Anti-human IgD APC (clone IA6-2) | Biolegend | Cat#348222 | 1:100 |
| PE-Streptavidin | ThermoFisher | Cat#SA10044 | 1:80 |
| PE/Cy7-Streptavidin | Biolegend | Cat#405206 | 1:80 |
| **Follicular helper T cell (Tfh) cytometry panel** | | | |
| Anti-human CD3 FITC (clone HIT3a) | BD Bioscience | Cat#555339 | 1:20 |
| Anti-human CD4 BV605 (clone RPA-T4) | BD Bioscience | Cat#562658 | 1:100 |
| Anti-human CD183 PE (clone 1C6) | BD Bioscience | Cat#557185 | 1:20 |
| Anti-human CD196 APC (clone G034E3) | Biologend | Cat#353416 | 1:100 |
| Anti-human CXCR5 BV711 (clone RF8B2) | BD Bioscience | Cat#740737 | 1:125 |
| Anti-human PD-1 PE/Cy7 (clone EH12.1) | BD Bioscience | Cat#561272 | 1:100 |
| **Critical commercial assays** | | | |
| Zombie UV Fixable Viability Kit | Biolegend | Cat#423107 | 1:400 |
| CFSE Cell Division Tracker Kit | Biolegend | Cat#423801 | NA |
| ELISA MAX^TM^ standard Set Human IFN-γ | Biolegend | Cat#430101 | NA |
| PMA | PeproTech | Cat#1652981 | NA |
| Ionomycin Calcium Salt | PeproTech | Cat#5608212 | NA |
| BFA | PeproTech | Cat#2031560 | NA |

**Table E2. Key Resources**

FITC, Fluorescein isothiocyanate; BV, Brilliant Violet; PMA, Phorbol 12-myristate 13-acetate; BFA, Brefeldin A; NA, not available

**Reference**

1. China NHCotPsRo. COVID-19 Treatment Guidelines, 8th edition. 2020.

2. Asthma GIf. Global Strategy for Asthma Management and Prevention 2020. June 8. Available from: https://ginasthma.org/.

3. Coverstone AM, Seibold MA, Peters MC. Diagnosis and Management of T2-High Asthma. The journal of allergy and clinical immunology In practice. 2020 Feb;8(2):442-450. eng. Epub 2020/02/11. doi:10.1016/j.jaip.2019.11.020. Cited in: Pubmed; PMID 32037108.

4. Halken S, Larenas-Linnemann D, Roberts G, Calderón MA, Angier E, Pfaar O, Ryan D, Agache I, Ansotegui IJ, Arasi S, Du Toit G, Fernandez-Rivas M, Geerth van Wijk R, Jutel M, Kleine-Tebbe J, Lau S, Matricardi PM, Pajno GB, Papadopoulos NG, Penagos M, Santos AF, Sturm GJ, Timmermans F, van Ree R, Varga EM, Wahn U, Kristiansen M, Dhami S, Sheikh A, Muraro A. EAACI guidelines on allergen immunotherapy: Prevention of allergy. Pediatric allergy and immunology : official publication of the European Society of Pediatric Allergy and Immunology. 2017 Dec;28(8):728-745. eng. Epub 2017/09/14. doi:10.1111/pai.12807. Cited in: Pubmed; PMID 28902467.

5. Nie J, Li Q, Wu J, Zhao C, Hao H, Liu H, Zhang L, Nie L, Qin H, Wang M, Lu Q, Li X, Sun Q, Liu J, Fan C, Huang W, Xu M, Wang Y. Quantification of SARS-CoV-2 neutralizing antibody by a pseudotyped virus-based assay. Nature protocols. 2020 Nov;15(11):3699-3715. eng. Epub 2020/09/27. doi:10.1038/s41596-020-0394-5. Cited in: Pubmed; PMID 32978602.

6. Liu L, Wang P, Nair MS, Yu J, Rapp M, Wang Q, Luo Y, Chan JF, Sahi V, Figueroa A, Guo XV, Cerutti G, Bimela J, Gorman J, Zhou T, Chen Z, Yuen KY, Kwong PD, Sodroski JG, Yin MT, Sheng Z, Huang Y, Shapiro L, Ho DD. Potent neutralizing antibodies against multiple epitopes on SARS-CoV-2 spike. Nature. 2020 Aug;584(7821):450-456. eng. Epub 2020/07/23. doi:10.1038/s41586-020-2571-7. Cited in: Pubmed; PMID 32698192.
